# Prevalence and Distribution of HPV Genotypes in Patients With Genital Warts: A Cross‐Sectional Study From South of Iran

**DOI:** 10.1002/hsr2.71345

**Published:** 2025-10-06

**Authors:** Maryam Sadat Sadati, Soodeh Jahangiri, Ahmadreza Azizi, S. Yasamin Parvar, Mojgan Akbarzadeh Jahromi, Yasamin Dehghan

**Affiliations:** ^1^ Department of Dermatology, School of Medicine Shiraz University of Medical Sciences Shiraz Iran; ^2^ Molecular Dermatology Research Center, School of Medicine Shiraz University of Medical Sciences Shiraz Iran; ^3^ Endocrine Research Center, Institute of Endocrinology and Metabolism Iran University of Medical Sciences Tehran Iran; ^4^ Student Research Committee Shiraz University of Medical Sciences Shiraz Iran; ^5^ Department of pathology, Maternal‐fetal medicine research center, School of medicine Shiraz University of medical sciences Shiraz Iran

**Keywords:** genital warts, genotype distribution, human papillomavirus (HPV), Iran, vaccination

## Abstract

**Background and Aims:**

Human papillomavirus (HPV), the most prevalent sexually transmitted infection, causes genital warts primarily through low‐risk genotypes. However, the presence of high‐risk genotypes highlights the need for genotype‐specific surveillance to guide clinical management and prevention strategies.

**Methods:**

This cross‐sectional study aimed to investigate the prevalence and distribution of HPV genotypes among patients diagnosed with genital warts at Shahid Faghihi Hospital in Shiraz, Iran, from 2017 to 2024. HPV genotyping was performed using PCR, and statistical analyses were used to evaluate associations between genotypes, sex, and age groups.

**Results:**

The study included 291 patients, predominantly female (85.2%) and aged 20–34 years (58.08%). Low‐risk HPV genotypes accounted for 91.35% of cases, while high‐risk genotypes HPV‐16 and HPV‐18 were detected in 6.92% and 1.73% of cases, respectively. A significant association was observed between sex and genotype distribution (*p* = 0.003), with high‐risk genotypes more prevalent in males (18.6%) than females (6.8%). No significant differences were found across age groups.

**Conclusion:**

Low‐risk HPV genotypes were most common in genital warts (91.35%), and HPV genotype distribution was significantly different between female and male patients. These findings emphasize the need for HPV vaccination programs and routine genotyping to guide clinical management in Iran's population.

## 1 Introduction

Human papillomavirus (HPV) infection is the primary cause of genital warts, or condyloma acuminata, the most common sexually transmitted infection (STI) worldwide [1]. More than 200 HPV genotypes have been identified, with approximately 40 of these affecting the anogenital tract [2]. Genital warts are primarily caused by low‐risk or non‐oncogenic genotypes, most commonly HPV types 6 and 11 [3]. High‐risk HPV types such as HPV‐16 and HPV‐18 are primarily associated with anogenital and oropharyngeal cancers [4]. Although less common in anogenital warts, these high‐risk types may still be present either as single infections or co‐infections with low‐risk types [5].

Prophylactic HPV vaccination has significantly reduced the incidence of genital warts and HPV‐related cancers in high‐coverage regions [6]. Quadrivalent and nonavalent HPV vaccines protect both low‐risk and high‐risk genotypes [7]. However, HPV vaccination is not yet included in the national immunization program of Iran, public awareness remains low, and access to the HPV vaccine is through private purchase, which is costly and limits coverage [8, 9]. Screening for cervical cancer in Iran is not organized at the national level. Pap smear testing is available but conducted irregularly, and HPV DNA testing is not widely implemented [10]. This poses significant challenges for HPV control and highlights the need for epidemiological data to inform policy and preventive strategies.

Previous studies have evaluated HPV genotype distribution among women undergoing cervical cancer screening [11], but data specific to patients with genital warts remains limited. This study aimed to characterize the prevalence of HPV genotypes and serotypes among genital wart patients in Shiraz, Iran, over 8 years, with emphasis on demographic variations.

## 2 Methods

### 2.1 Study Participants

This cross‐sectional study was conducted on patients aged 18 years or older who presented consecutively to the dermatology outpatient clinic of Shahid Faghihi Hospital with a clinical diagnosis of anogenital warts, requiring PCR, from 2017 to 2024. The diagnosis was confirmed through direct examination by trained dermatologists and included lesions of the labia, vagina, cervix, perianal and anal area, penis, scrotum, and pubis. All included patients had a specimen collected for HPV testing and underwent PCR genotyping according to protocol, and written informed consent was obtained. Patients were excluded if PCR was not performed or was negative for HPV DNA, if they had non‐anogenital warts or lesions of uncertain or alternative etiology, or if they had received prior ablative or topical therapy within the preceding 2 weeks. This study was approved by the Ethics Committee of Shiraz University of Medical Sciences (approval code: [IR. SUMS. MED. REC.1403.442]).

### 2.2 Sample Collection

Sampling brushes were used for collecting specimens from anogenital wart lesions [12, 13]. We used preservation solutions to elute the collected samples and stored them at −20°C.

### 2.3 DNA Extraction and HPV Genotyping

DNA extraction was performed using the E.Z.N.A.® DNA Extraction Kit (Omega Bio‐tek, USA), following the manufacturer's instructions. HPV genotyping was done by the GenMark HPV Detection Kit for HPV types 6, 11, 16, and 18. PCR amplification and detection were performed according to the kit protocol, which included internal controls to ensure the accuracy of the results. Each run included positive and negative controls to verify assay reliability. Before PCR, the DNA quality was checked to confirm that the samples were suitable.

### 2.4 Sample Size

The sample size was estimated to ensure sufficient representation of both high‐risk and low‐risk HPV genotypes. For this purpose, we used the higher prevalence estimate of high‐risk HPV reported in a previous study by Chalabiani et al., which indicated a prevalence of 67.2% among HPV‐positive individuals [14]. This conservative estimate helps ensure adequate statistical power for detecting differences in genotype distribution.

To achieve 80% power and a margin of error of 5%, the sample size was calculated using the following formula:

$$n=\frac{\left(1-P\right)\times P\times Z_{\mathrm{power}}^{2}}{E^{2}}$$

Substituting the values:

$$n=\frac{\left(1-0.672\right)\times 0.672\times {\left(0.84\right)}^{2}}{{\left(0.05\right)}^{2}}\;\approx 62.08$$

Therefore, the minimum required sample size was approximately 62 participants. A total of 291 patients were included in the study, exceeding the calculated requirement which allowed for subgroup comparisons and robust analysis.

### 2.5 Statistical Analysis

Statistical analysis was performed using SPSS Statistics for Windows, Version 25.0 (IBM Corp., 2017, Armonk, NY). Clinical and laboratory data were entered into an Excel sheet using study codes only, with names and other identifiers removed to maintain confidentiality. Data are presented as mean ± standard deviation (SD) or number (%). Between‐group comparisons were made using the *χ* ^2^ test or Fisher's exact test, as appropriate. A two‐sided *p*‐value < 0.05 was considered significant for all tests.

## 3 Results

The study included 291 patients with a mean age of 32.7 ± 10.5 years (range: 13–65 years). Most participants were female (85.2%, *n* = 248) and aged 20–34 years (58.1%, *n* = 169). Among 289 genotyped samples, low‐risk HPV genotypes (predominantly HPV‐6/11) were identified in 91.4% (*n* = 264) of cases, while high‐risk types HPV‐16 and HPV‐18 were detected in 6.9% (*n* = 20) and 1.7% (*n* = 5) of cases, respectively.

A significant association was found between sex and HPV genotype distribution (*p* < 0.001) (Table 1). HPV‐18 was exclusively found in females (*n* = 5), while HPV‐16 was more frequent in males (18.6% vs. 4.8%). No significant age‐based differences were observed (*p* > 0.05) (Table 2). Low‐risk genotypes dominated across all age groups (86.7%–100%); however, the highest frequency of high‐risk types was observed in the 35–49 age range (13.2%, *n* = 9/68). The lower rates of high‐risk HPV in the < 20 years (3.7%) and ≥ 50 years (0%) groups may be partially influenced by their smaller sample sizes. Overall, the data did not confirm a distinct age‐based pattern in high‐risk HPV distribution.

**Table 1:** Distribution of HPV genotypes by sex.

Genotype category	Female *n* (%)	Male *n* (%)	*p* value
Common types (6, 11)	229 (93%)	35 (81.4%)	0.003^a^
HPV‐16	12 (4.8%)	8 (18.6%)	
HPV‐18	5 (2%)	0	
High‐risk (HPV‐16 or 18)	17 (6.8%)	8 (18.6%)	0.018
Low‐risk (HPV‐6 or 11)	231 (93.1%)	35 (81.4%)	

^a^ *p* value calculated from the Chi‐square test comparing the distribution of all HPV genotypes between males and females.

**Table 2:** Distribution of HPV genotypes by age group.

Genotype category	< 20 years *n* (%)	20–34 years *n* (%)	35–49 years *n* (%)	≥ 50 years *n* (%)	*p* value
Common types (6,11)	26 (96.3%)	152 (91%)	59 (86.7%)	27 (100%)	0.375
HPV‐16	1 (3.7%)	11 (6.5%)	8 (11.7%)	0	
HPV‐18	0	4 (2.3%)	1 (1.4%)	0	
High‐risk (HPV‐16 or 18)	1 (3.7%)	15 (8.8%)	9 (13.2%)	0	0.155
Low‐risk (HPV‐6 or 11)	26 (96.3%)	154 (91.1%)	59 (86.7%)	27 (100%)	

## 4 Discussion

The present study investigated the epidemiologic characteristics and genotype distribution of HPV among patients with genital warts. Common low‐risk HPV types (predominantly HPV‐6/11) were found to be the most common (91.35%), and high‐risk types of HPV‐16 and −18 were less frequent. The majority of patients were female (85.2%), and our findings revealed significant differences in genotype and serotype distribution between male and female patients. Most participants were aged 20–34 years (58.08%), with only 9.28% being below 20 years or over 50 years. However, no age‐specific differences were found in our study.

### 4.1 Prevalence of HPV Types

Low‐risk genotypes were the most commonly identified, which is in line with the known patterns [15]. In a study among men with external genital lesions, low‐risk HPV types were detected in 73.2% of cases [16]. Similarly, Moossavi et al. studied women with genital warts in South Khorasan, Iran, and reported that low‐risk genotypes were present in 87.4% of the cases [17]. The authors report that HPV‐6 was the most common low‐risk type (40.71%), and HPV‐16 and HPV−18 were seen in 5.26% and 1.78% of the patients, respectively [17]. In another study of 250 cases with HPV‐positive genital warts in Beijing, China, 52.6% of participants had co‐infection with HPV‐6 and HPV‐11 [18]. These findings emphasize the predominance of low‐risk types in patients with genital warts; however, the presence of high‐risk types remains relevant due to their prognosis and oncogenic potential. Notably, the low‐risk types HPV‐6 and HPV‐11, along with high‐risk types such as HPV‐16 and HPV‐18, are covered by both the quadrivalent and nonavalent HPV vaccines, but not by the bivalent vaccine. In Iran, the locally produced HPV vaccine, Papilloguard®, is a bivalent formulation approved by the Iran Food and Drug Administration (IFDA), which covers HPV‐16 and HPV‐18, but provides no protection against HPV‐6 or HPV‐11. The quadrivalent formulation is currently unavailable.

### 4.2 Sex‐Related Patterns

Our data demonstrated a significant difference between males and females regarding HPV types, with high‐risk types found in 18.6% of male patients and 6.8% of female patients. Previous studies have reported varying results on this matter [19]. For instance, a study conducted in Guilan Province, Iran, among 122 patients (72.1% male) with HPV‐positive anogenital warts, found that HPV‐16 was significantly more prevalent among women [20]. This discrepancy may be due to the demographic and clinical characteristics of patients. Our participants referred to an outpatient center, while theirs included cases from both hospital and private laboratories. Differences in sexual behavior, HPV awareness, or healthcare access between geographic regions [21] as well as the low number of male participants in our study may have also contributed to these differences.

Similar to our findings, a recent study in Tehran, Iran, by Gholamzad et al. reported a significantly higher prevalence of high‐risk HPV genotypes in male participants compared to females [22]. In contrast, Al‐Awadhi et al. [23] observed no difference between the two sex groups. Another study with equal numbers of male and female participants (305 and 370, respectively) found that although low‐risk HPV types were significantly more common in male patients, the distribution of high‐risk types was similar across the two groups [24].

Behavioral differences, such as a higher number of lifetime sexual partners among men, can increase both exposure to HPV and the risk of persistent infection [25]. Additionally, anatomical distinctions in the male genital tract, such as the presence of keratinized epithelium and the absence of a mucosal environment, may affect viral acquisition and clearance, potentially leading to higher rates of high‐risk HPV persistence in men [26]. However, there are no comprehensive studies focused on these gender‐specific dynamics in the Iranian population; further research is needed to understand these patterns fully. The observed higher prevalence of high‐risk genotypes in our male patients, despite their smaller sample size, may also reflect different attitudes toward healthcare utilization. While women attend routine gynecologic exams, men may only seek care when symptomatic [27]. This discrepancy could lead to a higher burden of undiagnosed high‐risk HPV in males and warrants greater focus on screening and vaccination among men. Studies have shown that HPV‐related knowledge and willingness to receive the vaccine are low among men [28]. In a survey of college students in China, only 53.7% of male participants had heard of the HPV vaccine, compared to 78.3% of females. Additionally, 55.9% of males were willing to get vaccinated, compared to 80.4% of females (*p* < 0.001) [29]. Additionally, a cross‐sectional evaluation of Gardasil® use in Iran revealed that only 14.1% of recipients received the vaccine with correct dosing and indication, with the majority being women [30]. There is an urgent need for national‐level policy interventions and equitable access to HPV vaccines. Also, the lower knowledge and vaccination rates among men are alarming. We found that male participants had a higher percentage of high‐risk genotypes. High‐risk infections in men can serve as a reservoir for transmission to female partners, contributing to persistent infections and increasing the risk of cervical intraepithelial neoplasia and cancer. When these infections go undetected, they weaken the impact of prevention strategies that focus only on women. Integrating men into awareness campaigns and promotion of vaccination and condom use is essential to reduce the burden of HPV‐related diseases across populations [31].

### 4.3 Age‐Related Patterns

The majority of our participants were between 20 and 34 years of age, followed by the 35–49 age group. This trend aligns with the existing literature associating peak HPV acquisition following sexual debut [32]. A previous investigation in the Iranian population showed a high burden of HPV infection among those aged 30–44, peaking at 30–32 years [33].

In our study, the most prevalent HPV genotypes remained common across all age groups, and there was no association between different age groups and the genotype and serotypes of HPV. However, age‐specific patterns in genotype distribution have been noted in other studies. Zhu et al. reported that low‐risk HPV was most frequent among those aged 15–19 and 55–59, while high‐risk HPV peaked in the 55–59 age group [19].

While our study did not find age‐specific differences, the high proportion of cases occurring in young adults supports early vaccination strategies and the potential value of catch‐up vaccination in sexually active individuals who remain unvaccinated.

### 4.4 Public Health and Policy Implications

Economic models in low‐ and middle‐income countries show that vaccinating girls alone is generally the most cost‐effective strategy, while adding boys increases costs with smaller incremental benefits [34]. In Iran, prioritizing girls is likely most feasible; however, targeted male vaccination, especially in high‐risk groups, could reduce wart burden and high‐risk genotype transmission [34]. Also, incorporating HPV genotyping into national screening programs can significantly enhance early detection of high‐risk infections and enable more tailored follow‐up [35].

Cultural and systemic barriers limit HPV vaccine uptake in both males and females in Iran. These include ow awareness, misconceptions about who should be vaccinated, cost, and social stigma surrounding sexual activity [36, 37]. These challenges are further compounded by the lack of inclusion of the vaccine in the national immunization program and inadequate public education. It is crucial to expand the national coverage, reduce the financial barriers, and implement culturally sensitive awareness campaigns targeting both males and females to overcome these challenges [38].

### 4.5 Limitations

The present study was cross‐sectional, which limits the ability to investigate temporal or causal relations, and there was no follow‐up investigation. Additionally, our analysis did not account for the detection of multiple concurrent HPV infections, which should be taken into consideration when interpreting the results. Furthermore, due to limitations in our data, we were unable to differentiate low‐risk genotypes within the common HPV category.

A key limitation of this study is that all participants had clinically diagnosed genital warts, which are most often caused by low‐risk HPV types. Nevertheless, our study data provide important local epidemiological insights, help inform HPV vaccine selection, and highlight the occurrence of high‐risk types in wart cases.

## 5 Conclusions

This study examined the distribution of HPV genotypes among patients with genital warts in Shiraz, Iran. Low‐risk types were most prevalent, but high‐risk genotypes were also present, particularly among male patients. Most cases occurred in young adults, supporting the importance of early and catch‐up vaccination. The findings also underscore the importance of expanding HPV vaccination and awareness efforts to the broader population, ensuring that both men and women are equally informed and protected.

## Author Contributions

**Maryam Sadat Sadati:** methodology, supervision, formal analysis, conceptualization. **Soodeh Jahangiri:** writing – original draft, data curation. **Ahmadreza Azizi:** data curation, investigation. **S. Yasamin Parvar:** data curation, investigation. **Mojgan Akbarzadeh Jahromi:** resources, validation. **Yasamin Dehghan:** writing – review and editing, methodology, supervision, formal analysis.

## Ethics Statement

The ethical review committee of Shiraz University of Medical Sciences approved the study.

## Conflicts of Interest

The authors declare no conflicts of interest.

## 1 Transparency Statement

Dr. Yasamin Dehghan, author, affirms that this manuscript is an honest, accurate, and transparent account of the study being reported; that no important aspects of the study have been omitted; and that any discrepancies from the study as planned (and, if relevant, registered) have been explained.

## Data Availability

The data that support the findings of this study are available on request from the corresponding author. The data are not publicly available due to privacy or ethical restrictions. The data that support the findings of this study are available from the corresponding author upon reasonable request.
